# Joint Power and Multiple Access Control for Wireless Mesh Network with Rose Projection Method

**DOI:** 10.1155/2014/352809

**Published:** 2014-04-16

**Authors:** Meiqin Tang, Lili Shang, Yalin Xin, Xiaohua Liu, Xinjiang Wei

**Affiliations:** ^1^School of Mathematics and Statistics Science, Ludong University, Yantai 264025, China; ^2^School of Mechatronic Engineering and Automation, Shanghai University, Shanghai 270072, China

## Abstract

This paper investigates the utility maximization problem for the downlink of the multi-interface multichannel wireless mesh network with orthogonal frequency division multiple access. A cross-layer joint power and multiple access control algorithm are proposed. Rosen projection matrix is combined with Solodov projection techniques to build a three-memory gradient Rosen projection method, which is applied to solve this optimization problem. The convergence analysis is given and simulations show that the proposed solution achieves significant throughput compared with existing approaches.

## 1. Introduction

Both the existing standards and future development trends of broadband wireless communication system make their way forwards to resource allocation policy based on orthogonal frequency division multiple access (OFDMA). In OFDMA technology, each user's effective time and frequency resources are orthogonal to each other in radio link, which can avoid the interference between users and the effect produced by multipath attenuation. For the fixed portable applications, wireless channel is slowly changing. The essential difference of OFDMA technology and other multiple access technologies is that OFDMA can realize multiuser diversity in frequency selective fading channel and at the same time take advantage of the time difference in various channels. As an important part of the new generation of internet technology, wireless mesh networks (WMN) have gradually got into the civil commercial development and application stage, providing last few miles connectivity. We consider the resource control algorithm for WMN-based OFDM. Assuming that all users share the same bandwidth, and all channel state information has been collected in the *BS* of multilink and all subcarrier wave attenuation channels, as a result, user scheduling and routing selective problem can be solved by way of a joint resolution.

The utility function and economic models have been introduced into networks since the publication of the seminal paper [1] by Kelly et al. in 1998. The utility in the models represents the degree of the user's satisfaction when it acquires certain amount of the resource and the price is the cost per unit resource which the user must pay for this resource. In [2], column generation with greedy pricing was used to maximize the minimum throughput among all flows, and larger cases are considered which compute nearly optimal solutions. A mathematical programming model and assignment algorithms are developed for minimizing the schedule length in adaptive power and adaptive rate link scheduling in spatial-TDMA wireless networks in [3]. The objective in [4] is to maximize the aggregate utility of traffic flows in a multihop wireless network, and the constraints are imposed both due to self-interference and minimum rate requirements. In [5], resource management and admission control schemes for renewable energy sources in wireless mesh network are proposed. The goal is to maximize the energy sustainability of the network.

In this paper we develop a utility maximization problem of joint power control and multiple access resource allocation in wireless mesh network multicarrier orthogonal frequency division. Dual decomposition is used to decouple problem into power control and the multihop orthogonal frequency division multiple access scheduling in the transport layer and the MAC/PHY layer, which adjusts base cohort arrival rate. On the other hand, scheduling decides the subcarrier link rate and network modulation rate distribution. After transporting layer problem decoupled into speed control problem and the MAC/PHY layer into channel perception and queue scheduling problem, the maximum utility function is given combined with the control station to the other nodes in the output rate. Rosen projection matrix is combined with the Solodov projection techniques, namely, constructing a hyper plane using a point, which separates the current iteration point and the optimal solution of the problem sets. And then the current iteration points in this hyper plane projection, which structures a point list and gets set distance single drop of the optimal solution, thereby establish three-hybrid term memory gradient projection algorithm [6–10]. The convergence of the proposed algorithm is also given in this paper.

## 2. System Model

In this section, the network architecture and radio propagation model are first given, and then the elaborate network utility maximization model is given. The transmission is assumed in a time slot, the channel state information to maintain effective, but each data flow transmission is arbitrary and independent. Each relay node is equipped with a radio interface to accomplish the same time, send, and receive signals. Therefore, sending and receiving the subcarrier can be simultaneously conducted, and only one subcarrier can transmit data along the specified channel.

The network is considered as a flow set *φ* (*φ* = {*s* : *s* = 1,2,…, *S*}) from the source node to the base station *w*_*s*_ (*s* ∈ *φ*). Each flow transmits from the source node to the destination node path *L*_*s*_ with rate *v*_*s*_, where path *L*_*s*_ is the set from the source node to destination node pathway, expressed as *j* ∈ *L*_*s*_. The link capacity vector is expressed as *C* = {*c*_*ij*_} (*c*_*ij*_ is the channel capacity of the link) which is determined by the scheduling scheme. Scheduling scheme is connected by a set of orthogonal frequency division multiple cross-sub-carrier *Ω* = {*k* : *k* = 1,2,…, *K*}. The node transmit power is expressed as *P*_*i*_.

The noise on the link *ij* is *r*_*ij*_^*k*^, which can be expressed as

(1) $$\begin{array}{c} r_{ij}^{k}=\frac{\left|R_{ij}^{k}\right|}{H}, \end{array}$$

where *H* represents the noise power density and *R*_*ij*_^*k*^ is channel gain which is determined by path loss, disorder, and decay. We think that adaptive rate can allocate a limited set of modulation rate *V* = {0,1, 2,…, *N*}. The corresponding subcarrier *k* in the link *ij* transmission rate can be expressed as

(2) $$\begin{array}{c} c_{ij}^{k}=\min\left\{\left\lfloor \log_{2}\left(1+r_{ij}^{k}\right)\right\rfloor ,N\right\}. \end{array}$$

The wireless mesh network from a gateway to the source node is composed of *n* nodes. If the distance of adjacent nodes is *D*, each node of the required data flow is *σ*_*i*_. The actual arrival of the maximum data flow is *ζ*_*i*_, and assume that the actual data traffic on the link is *F*_*i*_. We set

(3) $$\begin{array}{c} \frac{\zeta_{i}}{\sigma_{i}}=\frac{\zeta_{j}}{\sigma_{j}} \end{array}$$

which is to ensure the fairness of all node data flow. The network throughput is

(4) $$\begin{array}{c} \zeta =\overset{n}{\underset{i=1}{\sum }}\phi_{i}. \end{array}$$

Nodes access network in a similar way with the MAC protocol in CSMA/CA. When the destination node receives the ACK packets from the source node, a data transmission is finished. Each node transmitting data power is *P*, *P* ∈ [0, *P*_max⁡_], and *P*_max⁡_ is the maximum of power. *M*(*d*) is the path transmission gain, *τ* is the path attenuation index, *d* is the data transmission distance, *f*_*C*_ is carrier, and *c* is light speed; channel attenuation can be expressed as

(5) $$\begin{array}{c} M\left(d\right)=\frac{1}{{\left(4\pi f_{c}/c\right)}^{2}d^{\tau }}. \end{array}$$

If each node in wireless mesh network can assembly *K* interface, and the network has *N* orthogonal channel available, and the data can only be transmitted in adjacent nodes, each node can detect whether the adjacent nodes send data, then

(6) $$\begin{array}{c} P_{\min}M\left(D\right)>CS_{\text{TH}}. \end{array}$$

Each node transmitting data power is *P*, and the power constraint is

(7) $$\begin{array}{c} P\geq \frac{CS_{\text{TH}}}{M\left(D\right)},\;\;P\leq P_{\max}, \end{array}$$

if *R* = {*r*_*BS*_^*s*^ ≥ 0 : *s* ∈ *φ*} is long-term average arrival rate to the base station set. Definite Ψ = {*C* : *C* = [*c*_1_, *c*_2_,…, *c*_*ij*_,….]} as all feasible line capacity vector collection, and *F* = {*f*_*ij*_^(*s*)^ ≥ 0 : *s* ∈ *φ*, *ij* ∈ *L*_*S*_} expresses connection set, where *f*_*ij*_^(*s*)^ represents *ij* link to *s* power capacity.

The utility *U*_*s*_ in the models represents the degree of the user's satisfaction with service quality and cost in terms of resource consumption, which are continuously differentiable nonlinear functions. The optimization problem is formulated as follows:

(8) $$\begin{array}{c} \underset{R,f,C}{\max}\underset{s:n=BS}{\sum }\left(v_{n}^{\left(s\right)}\lambda P\right) \end{array}$$

s.t.

(9) $$\begin{array}{c} P\leq P_{\max},\;\;P\geq \frac{CS_{\text{TH}}}{M\left(D\right)} \end{array}$$

(10) $$\begin{array}{c} v_{n}^{\left(s\right)}+\underset{i:in\in L_{s}}{\sum }f_{in}^{\left(s\right)}\leq \underset{i:nj\in L_{s}}{\sum }f_{nj}^{\left(s\right)},\;\forall s,\;\;n\neq w_{s} \end{array}$$

(11) $$\begin{array}{c} f_{ij}=\underset{s:ij\in L_{s}}{\sum }f_{nj}^{\left(s\right)}\leq C_{ij},\;\forall ij \end{array}$$

(12) $$\begin{array}{c} f_{ij}^{\left(s\right)}=0,\;\text{if}\;\;ij\notin L_{s}\;\forall s,ij \end{array}$$

(13) $$\begin{array}{c} C\in \Psi , \end{array}$$

where *λ* is the weighted factor, which can improve the speed and power of fairness and eliminate the dimension. Equation (10) can ensure that the system reaches the objective of the total efficiency; if *n* ≠ *BS*, then

(14) $$\begin{array}{c} v_{n}^{\left(s\right)}=0, \end{array}$$

otherwise,

(15) $$\begin{array}{c} \underset{i:in\in L_{s}}{\sum }f_{in}^{\left(s\right)}=0. \end{array}$$

Equation (11) is the rate constraint. And (12) expresses that if the signal is without the link *ij*, the link rate is 0; and (13) expresses the link capacity constraint vector in the feasible region.

## 3. The Proposed Algorithm-Based Mixing Three-Term Memory Gradient Projection Algorithm

Assume

(16) $$\begin{array}{c} x=\left(v_{n}^{\left(s\right)},\lambda P\right). \end{array}$$

The original problem

(17) $$\begin{array}{c} \underset{R,f,C}{\max}\underset{s:n=BS}{\sum }\left(v_{n}^{\left(s\right)}\lambda P\right) \end{array}$$

can be translated into

(18) $$\begin{array}{c} \underset{R,f,C}{\min}-\underset{s:n=BS}{\sum }U_{s}\left(x\right). \end{array}$$

Assume the utility function of variables in the feasible domain of *R*,

(19) $$\begin{array}{c} R=\left\{x\in E^{n}\;|\;-\underset{s:n=BS}{\sum }U_{sj}\left(x\right)\leq 0,j\in L=\left\{1,2,\ldots ,s+1\right\}\right\}, \end{array}$$

where

(20) $$\begin{array}{c} -\underset{s:n=BS}{\sum }U_{s}\left(x\right) \end{array}$$

is assumed as a nonlinear function:

(21) $$\begin{array}{c} g\left(x\right)=-\nabla \left(-\underset{s:n=BS}{\sum }U_{s0}\left(x\right)\right)\\ a_{j}\left(x\right)=\nabla \left(-\underset{s:n=BS}{\sum }U_{s0}\left(x\right)\right),\;j\in L. \end{array}$$

The index set *j* ∈ *L*, |*J*| indicates the *J* index number, and note that

(22) $$\begin{array}{c} A\left(x\right)=\left(a_{j}\left(x\right),j\in J\right)=A_{J}\\ J_{\delta }\left(x\right)=\left\{j\in L\;|\;-\left(-\underset{s:n=BS}{\sum }U_{sj}\left(x\right)\right)\leq \delta \right\}. \end{array}$$

Starting from point *x* ^*k*^, take the function *x* ^*k*^ at the fastest decline in direction as the search direction [10].

First, combine Rosen projection matrix with the Solodov projection techniques to construct a hyper plane, which can separate the current iteration and get the optimal solution of the proposed problem. Secondly, project the current iteration in the hyper plane, which constructs a sequence of points. Then the optimal solution can be obtained as a set distance of single point drop. Thereby establishing the solution of linear or nonlinear constrained optimal problem is converted to three-hybrid term memory gradient projection algorithm.

Assume *M* as the K-T point set of the problem of min⁡_*R*,*f*,*C*_ − ∑_*s*:*n*=*BS*_ *U*_*s*_(*x*), which satisfies the *M* condition mentioned:

(3) ∇∑s:n=BSUs(x)+ξ1∇(P−Pmax⁡)+ξ2∇(CSTHG(D)−P) +ξ3∇(rn(s)+∑i:in∈Lsfin(s)≤∑j:nj∈Lsfnj(s)) +ξ4∇(∑s:ij∈LSfij(s)−Cij)=0fij(s)=0, if,  ij∉Ls  ∀s,ijC∈ψ,

where *ξ*_*i*_,  *i* = 1,2, 3,4 is real number.

**Lemma 1**

If *x* ^*k*^ ∈ *R* is the non-K-T point of

(24) $$\begin{array}{c} \underset{R,f,C}{\min}-\underset{s:n=BS}{\sum }U_{s}\left(x\right), \end{array}$$

where *β*_*k*_^1^ meets (4), (5) and *β*_*k*_^2^ meets (6), (7), and (8), then

(25) $$\begin{array}{c} ||S_{k}^{2}||\leq \left(1+\frac{1}{\Delta_{1}}+\frac{1}{\Delta_{2}}\right)||P_{J_{k}}\left(x^{k}\right)g\left(x^{k}\right)||. \end{array}$$

**Lemma 2**

If *x* ^*k*^ ∈ *R* is the non-K-T point of

(26) $$\begin{array}{c} \underset{R,f,C}{\min}-\underset{s:n=BS}{\sum }U_{s}\left(x\right), \end{array}$$

where *β*_*k*_^1^ meets (3), (4), and (5) and *β*_*k*_^2^ meets (8), (9), and (10), then

(27) $$\begin{array}{c} g{\left(x^{k}\right)}^{T}S_{k}^{2}\geq \overset{2}{\underset{r=1}{\prod }}\frac{1+\Delta_{r}}{2+\Delta_{r}}{||P_{J_{k}}\left(x^{k}\right)g\left(x^{k}\right)||}^{2}. \end{array}$$

**Proof**

By the definition of *S*_*k*_^1^ and (1), we can get

(28) gkTSk1=gkTPJk(xk)(gk+βk1dk−1)≥||PJk(xk)gk||2−|βk1gkPJk(xk)dk−1|≥||PJk(xk)gk||2−11+Δ1gkTSk1.

Based on

(29) $$\begin{array}{c} g^{kT}S_{k}^{1}\geq \frac{1+\Delta_{1}}{2+\Delta_{1}}\cdot {||P_{J_{k}}\left(x^{k}\right)g^{k}||}^{2}, \end{array}$$

then by the definition and (2), we have

(30) $$\begin{array}{c} g^{kT}S_{k}^{2}\geq \frac{1+\Delta_{2}}{2+\Delta_{2}}\cdot g^{kT}S_{k}^{1}\geq \overset{2}{\underset{r=1}{\prod }}\frac{1+\Delta_{r}}{2+\Delta_{r}}{||P_{J_{k}}\left(x^{k}\right)g^{k}||}^{2}. \end{array}$$

Assuming that

(31) $$\begin{array}{c} \varphi_{i}\left(\cdot \right):E_{+}^{1}E_{+}^{1}\;\left(i=1,2\right), \end{array}$$

is two continuous functions:

(32) $$\begin{array}{c} \varphi_{i}\left(\lambda \right)=0⟹\lambda =0,\;i=1,2, \end{array}$$

(33) $$\begin{array}{c} E_{+}^{1}=\left\{\lambda \;|\;\lambda \geq 0,\lambda \in E^{1}\right\}, \end{array}$$

we set *ϕ*_1_(*x*) as nonnegative continuous functions of *R* and *ϕ*_2_(*x*) as right continuous function of *R*. The detailed steps of the proposed algorithm are as follows.

**Step 1**

Consider  ∀*x* ^0^ ∈ *R*, *δ*_0_ > 0, *β* > 1, Δ_1_ > 0, Δ_2_ > 0, *c* > 0, 0 < *τ* < 1, *d* ^0−1^ = *d* ^0−2^ = 0. Assume

(34) $$\begin{array}{c} k∶=0. \end{array}$$

**Step 2**

Set

(35) $$\begin{array}{c} J_{k}=J_{\delta_{k}}\left(x^{k}\right). \end{array}$$

If

(36) $$\begin{array}{c} \left|\det\left(A_{J_{k}}^{T}\left(x^{k}\right)A_{J_{k}}\left(x^{k}\right)\right)\right|\geq \delta_{k}, \end{array}$$

then go to Step 3; otherwise, set

(37) $$\begin{array}{c} x^{k+1}=x^{k},\\ \delta_{k}=\frac{\delta_{k}}{\beta },\;\;k∶=k+1. \end{array}$$

**Step 3**

Calculate *B*_*J*_*k*__, *P*_*J*_*k*__, *U*_*J*_*k*__^*k*^. If

(38) $$\begin{array}{c} P_{J_{k}}g^{k}=0,\;{\left(\mu_{J_{K}}^{K}\right)}^{T}\left(-\underset{s:n=BS}{\sum }U_{sJ_{k}}\left(x^{k}\right)\right)=0,\;\mu_{J_{K}}^{K}\geq 0, \end{array}$$

then stop, where *x* ^*k*^ is the K-T point of

(39) $$\begin{array}{c} \underset{R,f,C}{\min}\underset{s:n=BS}{\sum }U_{s}\left(x\right). \end{array}$$

Otherwise go to Step 4.

**Step 4**

(40)dk=(1+|μJkkTWJkT|)PJk(gk+∑r=12βkrdk−r)+βJkT ×[(1+|μJkkTWJkk|)VJkk−(gkTPJk(gk+∑r=12βkrdk−r)+μJkkTVJkk)WJkT],(4)VJkk=(Vjk,j∈Jk)T:Vjk={−φ1(−μjk),μjk≤0−φ2(μjk)−∑s:n=BSUsj(xk),−μjk>0WJkk=(Wjk,j∈Jk)T:Wjk={ϕ1(xk),j∈L1∩Jkϕ2(xk),j∈L2∩Jk,

where *β*_*k*_^1^ meets (3), (4), and (5) and *β*_*k*_^2^ meets (6), (7), and (8).

**Step 5**

Set *λ* ← 1; if

(42) $$\begin{array}{c} W_{j}^{k}=\{\begin{array}{cc} x^{k+1}=x^{k}+\lambda_{k}d^{k}\in R & \\ \nabla \left(-\underset{s:n=BS}{\sum }U_{s0}{\left(x^{k}+\lambda d^{k}\right)}^{T}\right)d^{k} & \\ \;\leq \gamma \nabla {\left(-\underset{s:n=BS}{\sum }U_{s0}{\left(x^{k}\right)}^{T}\right)}^{T}d^{k} & \end{array} \end{array}$$

then if

(43) $$\begin{array}{c} \lambda_{k}=\lambda ,\;\;y^{k}=x^{k}+\lambda d^{k} \end{array}$$

go to Step 6; otherwise, take

(44) $$\begin{array}{c} \lambda_{\text{new}}\in \left[\bar{\sigma_{1}}\lambda ,\bar{\sigma_{2}}\lambda \right],\;\lambda ⟵\lambda_{\text{new}}\;\;\;\;\;\;\;\;\;\; \end{array}$$

and go to Step 5.

**Step 6**

Set

(45) $$\begin{array}{c} J_{K}=J_{\delta_{k}}\left(y^{k}\right). \end{array}$$

If

(46) $$\begin{array}{c} \det\left(A_{J_{k}}^{T}\left(y^{k}\right)A_{J_{k}}\left(y^{k}\right)\right)\geq \delta_{k}, \end{array}$$

then calculate *B*_*J*_*k*__, *P*_*J*_*k*__, *μ*_*J*_*k*__. If

(47) $$\begin{array}{c} P_{J_{k}}g^{k}=0,\;\;{\left(\mu_{J_{k}}^{k}\right)}^{T}\left(-\underset{s:n=BS}{\sum }U_{sJ_{k}}\left(y^{k}\right)\right)=0,\;\mu_{J_{k}}^{k}\geq 0 \end{array}$$

then stop. *y* ^*k*^ is the K-T point of

(48) $$\begin{array}{c} \underset{R,f,C}{\min}-\underset{s:n=BS}{\sum }U_{s}\left(x\right), \end{array}$$

or else go to Step 7. If

(49) $$\begin{array}{c} \det\left(A_{J_{k}}^{T}\left(y^{k}\right)A_{J_{k}}\left(y^{k}\right)\right)\geq \delta_{k} \end{array}$$

is not established, we set

(50) $$\begin{array}{c} y^{k+1}=y^{k},\;\;\delta_{k}∶=\frac{\delta_{k}}{\beta }\delta ,\;\;k∶=k+1, \end{array}$$

and then go to Step 6.

**Step 7**

If

(51) $$\begin{array}{c} \nu_{k}=\nabla \left(-\underset{s:n=BS}{\sum }U_{s0}\left(y^{k}\right)\right),\\ {\bar{x}}_{k+1}=x^{k}-\frac{\left(\nu_{k},x^{k}-y^{k}\right)}{{||\nu_{k}||}^{2}}. \end{array}$$

Set

(52) $$\begin{array}{c} x^{k+1}={\bar{x}}_{k+1},\;k=k+1, \end{array}$$

and then go to Step 2.

**Lemma 3**

If *x* ^*k*^ ∈ *R* and meets the non K-T point of the

(53) $$\begin{array}{c} \underset{R,f,C}{\min}-\underset{s:n=BS}{\sum }U_{s}\left(x\right), \end{array}$$

then *d* ^*k*^ is the descent direction of

(54) $$\begin{array}{c} -\underset{s:n=BS}{\sum }U_{s0}\left(x\right) \end{array}$$

at *x* ^*k*^.

## 4. Convergence Analysis of the Proposed Algorithm

Set R* as the optimal solution set of

(55) $$\begin{array}{c} \underset{R,f,C}{\min}-\underset{s:n=BS}{\sum }U_{s}\left(x\right). \end{array}$$

Suppose R* is convex: the global convergence results are as follows.

**Theorem 4**

Located on

(56) $$\begin{array}{c} \left(-\underset{s:n=BS}{\sum }U_{s0}\left(\cdot \right)\right) \end{array}$$

is continuously differentiable pseudorandom function, and {*x* ^*k*^} A is produced by the algorithm (PTMG ) to produce infinite iterative sequence; then

(57) $$\begin{array}{c} \underset{k\to \infty }{\lim}x^{k}=x^{*}, \end{array}$$

where x* ∈ R*.

**Proof**

As we know, sequence {||*x* ^*k*^ − x*||} is monotonically decreasing. Therefore, x* is bounded; according to Lemma 1, we can get that {d*} is bounded. According to the definition of knowledge, {*y* ^*k*^} is bounded. So there exists *M* > 0 satisfying ∀*k* ∈ *N*, ||*v*_*k*_ ≤ *M*||. Thus, by the Step 7, we can get

(58) γ2λk2(∇f0(xk),dk)2M2≤λk2(∇f0(xk),dk)2M2 ≤(vk,xk−yk)2||vk||2≤||xk−x∗||2−||xk+1−x∗||2.

That is,

(59) γ2M2∑k=1∞λk2(∇f0(xk),dk)2 ≤∑k=1∞(||xk−x∗||2−||xk+1−x∗||2)<+∞.

Therefore,

(60) $$\begin{array}{c} \underset{k\to \infty }{\lim}\lambda_{k}^{2}\left(\nabla f_{0}\left(x^{k}\right),d^{k}\right)=0, \end{array}$$

and we get

(61) $$\begin{array}{c} \underset{k\to \infty }{\lim}\left(\nabla f_{0}\left(x^{k}\right),d^{k}\right)=0. \end{array}$$

Actually, if there exists *ε*_0_, an infinite subset *K* ∈ *N*, so that

(62) $$\begin{array}{c} \left(\nabla f_{0}\left(x^{k}\right),d^{k}\right)\leq -\varepsilon_{0}, \end{array}$$

we set

(63) $$\begin{array}{c} \underset{k\in K,k\to \infty }{\lim\;\;\inf}\lambda_{k}=\lambda . \end{array}$$

Then *λ* = 0, so there exists an infinite subset *k* ∈ *K* so that

(64) $$\begin{array}{c} \underset{k\in K,k\to \infty }{\lim\;\;\inf}\lambda_{k}=\lambda . \end{array}$$

The algorithm shows

(65) $$\begin{array}{c} \sigma_{1}\leq \rho_{k}\leq \sigma_{2},\;\psi_{k}=\frac{\lambda^{k}}{\rho^{k}}, \end{array}$$

so that

(66) $$\begin{array}{c} \left(\nabla f_{0}\left(x^{k}+\psi_{k}d^{k}\right),d^{k}\right)>\gamma \left(\nabla f_{0}\left(x^{k}\right),d^{k}\right),\;\forall k\in K. \end{array}$$

Suppose

(67) $$\begin{array}{c} \underset{k\in K,k\to \infty }{\lim}x^{k}=x^{*},\\ \underset{k\in K,k\to \infty }{\lim}d^{k}=d^{*}. \end{array}$$

There are

(68) $$\begin{array}{c} -\varepsilon_{0}\left(1-\gamma \right)\geq \left(\nabla f_{0}\left(x^{*}\right),d^{*}\right)\left(1-\gamma \right)\geq 0 \end{array}$$

which is in contradiction with

(69) $$\begin{array}{c} \gamma \in \left(0,1\right), \end{array}$$

and thus

(70) $$\begin{array}{c} \underset{k\to \infty }{\lim}\left(\nabla f_{0}\left(x^{k}\right),d^{k}\right)=0. \end{array}$$

Any point of {*x* ^*k*^} is the K-T point of

(71) $$\begin{array}{c} \underset{R,f,C}{\min}-\underset{s:n=BS}{\sum }U_{s}\left(x\right). \end{array}$$

Then by the pseudo convexity of the

(72) $$\begin{array}{c} \underset{R,f,C}{\min}-\underset{s:n=BS}{\sum }U_{s0}\left(x\right), \end{array}$$

we can see x* ∈ R*. {||*x* ^*k*^ − x*||} decreases monotonically limit exits. Therefore,

(73) $$\begin{array}{c} \underset{k\to \infty }{\lim}\left(x^{k}-x^{*}\right)=\underset{k\in K,k\to \infty }{\lim}x^{k}-x^{*}=0, \end{array}$$

and so the proposed problem

(74) $$\begin{array}{c} \underset{R,f,C}{\min}-\underset{s:n=BS}{\sum }U_{s}\left(x\right) \end{array}$$

is globally convergent.

## 5. Numerical Examples

In this section, we aim to show the effectiveness of the proposed algorithm. We uniformly distribute 200 nodes in a square area of dimensions 1000 m × 1000 m, where each node can select for the transmission across each link. *P*_max⁡_ is set to be 100 mW, and the path attenuation parameter *τ* is set to 4.

### 5.1. The Influence of Parameter *v*_*n*_^(*s*)^

**Figure 1:**
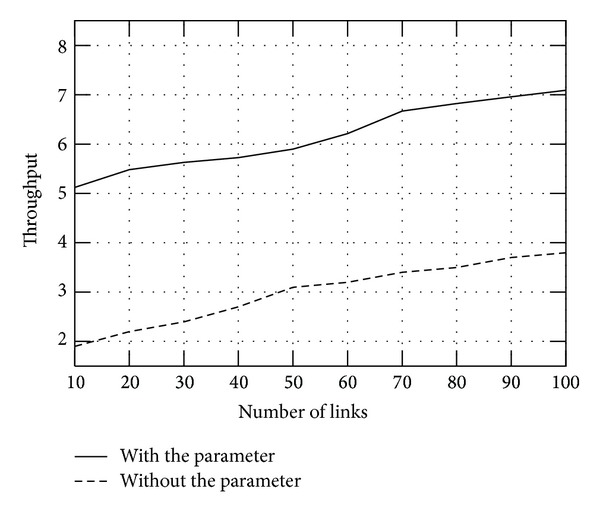
The influence of parameter *v*_*n*_^(*s*)^.

We first investigate the effect of *v*_*n*_^(*s*)^ on the optimal throughput versus links, and a specified number of links (L) is selected.  Figure 1 shows the attained network throughput with *v*_*n*_^(*s*)^ and without it, from which we can find that proposed model can get higher throughput than that without it. It is due to the adjustment of the parameter which can improve the system efficiency.

### 5.2. Fairness of the System

**Figure 2:**
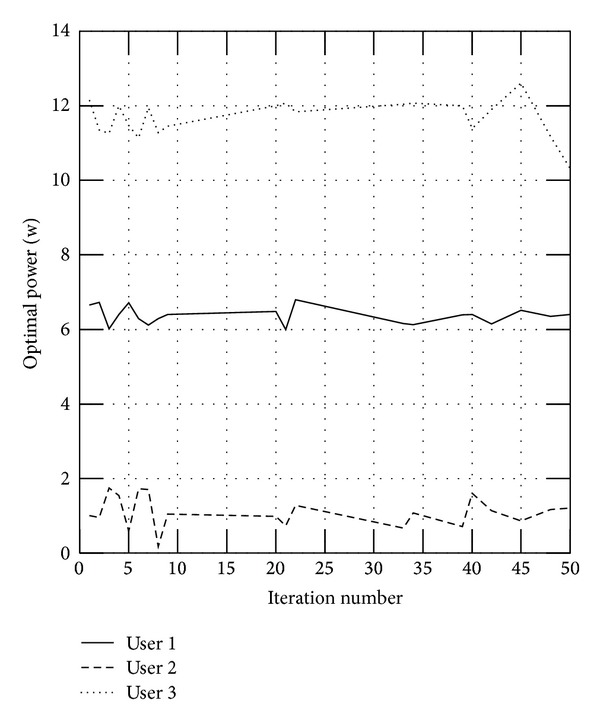
Optimal power without the parameter *λ*.

**Figure 3:**
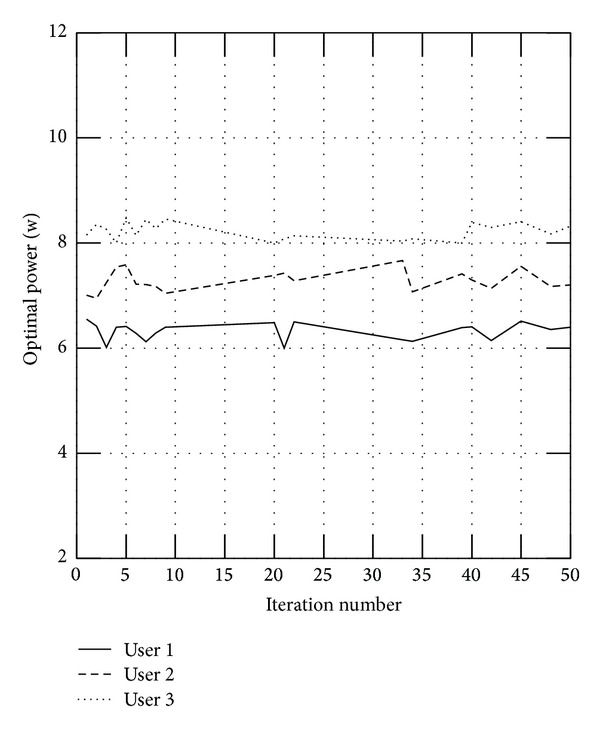
Optimal power with the parameter *λ*.

And we will also verify the influence of parameter *λ* for the users. We select three users randomly.  Figure 2 shows the users' powers without the fairness parameter, where user 2 is allocated less power while user 3 is allocated more power; and the fairness can be assured with the parameter, as shown in Figure 3.

### 5.3. Performance with Different Methods

**Figure 4:**
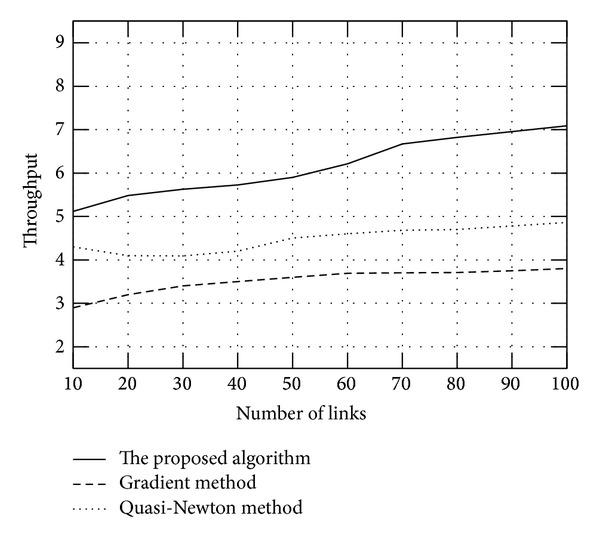
Throughput comparison with different methods.

We also show the throughput versus links with different methods in Figure 4, which adopt the same parameter of the system. From the figures we can find that the proposed algorithm can get better performance the algorithms solved with gradient method and Quasi-Newton method.

## 6. Conclusions

This work proposes to optimize joint power and multiple access control in wireless mesh network. Dual decomposition is used to decouple problem into power control and the multihop orthogonal frequency division multiple access scheduling in the transport layer and the MAC/PHY layer. Rosen projection matrix combined with the Solodov projection techniques is used to solve the proposed algorithm. Simulation studies show that the proposed algorithms are effective to solve the optimization problem and outperform the existing approaches in terms of throughput and the fairness of the users can be assured. The nonconvexity of the resource control problem is our future research work.

## References

[1] Kelly FP, Maulloo AK, Tan DKH (1998). Rate control for communication networks: shadow prices, proportional fairness and stability. *Journal of the Operational Research Society*.

[2] Luo J, Rosenberg C, Girard A (2010). Engineering wireless mesh networks: joint scheduling, routing, power control, and rate adaptation. *IEEE/ACM Transactions on Networking*.

[3] Hedayati K, Rubin I, Behzad A (2010). Integrated power controlled rate adaptation and medium access control in wireless mesh networks. *IEEE Transactions on Wireless Communications*.

[4] Kim TS, Yang Y, Hou JC, Krishnamurthy SV (2013). Resource allocation for QoS support in wireless mesh networks. *IEEE Transcations on Wireless Communications*.

[5] Cai LX, Liu YK, Luan TH (2014). Sustainability analysis and resource management for wireless mesh networks with renewable energy supplies. *IEEE Journal on Selected Areas in Communications*.

[6] Rosen JB (1960). The gradient projection method for nonlinear programming. Part I. Linear constraints. *Journal of the Society for Industrial and Applied Mathematics*.

[7] Rosen JB (1960). The gradient projection method for nonlinear programming. Part2, nolinear constraints. *Journal of the Society for Industrial and Applied Mathematics*.

[8] Solodov MV, Svaiter BF (1999). A hybrid projection-proximal point algorithm. *Journal of Convex Analysis*.

[9] Solodov MV, Svaiter BF (1999). A new projection method for variational inequality problems. *SIAM Journal on Control and Optimization*.

[10] Solodov MV, Svaiter BF (2000). A truly globally convergent Newton-type method for the monotone nonlinear complementarity problem. *SIAM Journal on Optimization*.

